# Effects of antenatal corticosteroids in twin neonates with late preterm birth (ACTWIN [Antenatal Corticosteroids in TWIN late preterm neonates] trial): study protocol for a randomized controlled trial

**DOI:** 10.1186/s12884-019-2235-5

**Published:** 2019-04-03

**Authors:** Subeen Hong, Seung Mi Lee, Dong Wook Kwak, Joongyub Lee, So Yeon Kim, Jeong Won Oh, Sohee Oh, Chan-Wook Park, Joong Shin Park, Jin Hoon Chung, Jong Kwan Jun

**Affiliations:** 1 Department of Obstetrics and Gynecology, Seoul National University College of Medicine, Seoul, Republic of Korea; 2 Department of Obstetrics and Gynecology, Seoul National University Bundang Hospital, Seongnam, Republic of Korea; 3 Department of Obstetrics and Gynecology, Cheil General Hospital and Women’s Healthcare Center, Dankook University College of Medicine, Seoul, South Korea; 4 Department of Obstetrics and Gynecology, Ajou University Medical School, Suwon, Korea; 5 Department of Prevention and Management, Inha University Hospital, Incheon, Korea; 6 School of Medicine, Inha University, Incheon, Korea; 7 Department of Biostatistics, Seoul Metropolitan Government Seoul National University Boramae Medical Center, Seoul, Korea

**Keywords:** Antenatal corticosteroids, Late preterm birth, Twin pregnancies, Respiratory morbidity, Randomized controlled trial

## Abstract

**Background:**

Antenatal corticosteroids have been proven to prevent adverse outcomes including respiratory morbidities in preterm neonates before 34 weeks of gestation. Recently, it has been suggested that antenatal corticosteroids may also be effective in singleton late preterm pregnancies, and guidelines recommend the use of corticosteroids in singleton pregnant women who are at risk for late preterm birth. On the contrary, there is a paucity of information regarding the effectiveness of corticosteroids in twin neonates with late preterm birth. This study aims to determine the effectiveness of antenatal corticosteroids in late preterm twin neonates.

**Methods:**

In this multicentre randomized controlled trial, women who are at risk for late preterm birth will be enrolled at 34 0/7 to 36 5/7 weeks of gestation. The participants will be randomly assigned to receive antenatal corticosteroids (betamethasone 12 mg, 3 mL intramuscularly [IM]) or placebo (normal saline 3 mL IM). The perinatal outcomes will be compared between the two groups of cases. The primary outcome is severe respiratory complications (the use of continuous positive airway pressure or high-flow nasal cannula for at least 12 h, supplemental oxygen administration with a fraction of oxygen 0.3 or more for at least 24 h, mechanical ventilation, or extracorporeal membranes oxygenation) or perinatal death within the first 72 h of delivery. The secondary outcomes are neonatal mortality and/or other neonatal morbidities.

**Discussion:**

This study will be the first randomized controlled trial that evaluates the effectiveness of antenatal corticosteroids in late preterm twin neonates.

**Trial registration:**

NCT03547791(ClinicalTrials.gov), first submitted date: March 29, 2018, first posted date: June 6, 2018 (retrospectively registered).

## Background

The number of multifetal pregnancies has recently been increasing. Between 1980 and 2009, the frequency of twin pregnancies in the United States increased by three-fourths, from 18.9 to 33.3 per 1000 births, and this phenomenon has been attributed to advancing maternal age and increased use of assisted reproductive technique [1]. Multifetal pregnancies are at higher risk for maternal and neonatal complications compared with singleton pregnancies, and preterm birth is one of the most important issues in twin pregnancies [2–5]. Indeed, the rate of preterm birth in twin pregnancy is as high as 50%, with most cases occurring in the late preterm period (34 0/7 to 36 6/7 weeks of gestation) [6, 7]. Late preterm birth has recently emerged as a major health problem, because late preterm neonates are at increased risk not only for neonatal morbidities but also for childhood complications as compared with term neonates [8–10].

For pregnant women at risk for early preterm delivery (before 34 weeks of gestation), administration of antenatal corticosteroids is a standard treatment to reduce perinatal mortality and morbidity, especially in terms of respiratory complications [11–13]. Antenatal corticosteroids also can be beneficial for neonates at risk for late preterm birth. Recently, a large randomized controlled trial was conducted to investigate the effectiveness of antenatal corticosteroids in singleton late preterm neonates [14]. This study reported that administration of betamethasone significantly reduced the rate of neonatal respiratory complications in singleton late preterm neonates. Based on this result, guidelines from the National Institute for Health and Care Excellence (NICE), Society for Maternal-Fetal Medicine (SMFM) and American College of Obstetricians and Gynecologists (ACOG) recommend administration of betamethasone for pregnant women at risk of late preterm birth in singleton gestation [15–17].

Some studies reported that antenatal corticosteroids also improved neonatal outcomes in twin preterm births before 34 weeks [13, 18, 19]. Based on these evidences and effectiveness of corticosteroids in preterm singleton pregnancy, ACOG recommends that one course of antenatal corticosteroids should also be used in multifetal pregnant women who are at risk of early preterm birth (before 33 6/7 weeks of gestation) [15, 20]. On the contrary, there is a paucity of information regarding the effectiveness of antenatal corticosteroids in twin late preterm birth and the guidelines are not established in this population [15].

The determination of the effectiveness of antenatal corticosteroids in twin late preterm birth is a critical issue with regard to several points. First, the neonatal morbidities in twin preterm neonates may be different from that in singleton preterm neonates [20]. Second, the results of some retrospective studies on the effectiveness of antenatal corticosteroids in preterm twin neonates were conflicting. Some studies reported that the effect of antenatal corticosteroids on neonatal outcomes in twin pregnancy was comparable with that in singleton pregnancy [13, 18, 19, 21]. However, there are disagreements as to the effect of antenatal corticosteroids in twin pregnancies [22, 23]. In addition, a recent report on the use of a corticosteroid in twin late preterm birth showed no reduction in respiratory morbidity [18]. Third, the pharmacokinetics in women with twin pregnancy after administration of betamethasone was different from that in singleton pregnant women, probably resulting in a different effectiveness of corticosteroids between singleton and twin pregnancies [24].

To determine this issue, we will evaluate the efficacy of antenatal corticosteroids in twin pregnancies that are at risk for late preterm birth.

## Methods

### Study design

**Fig. 1:**
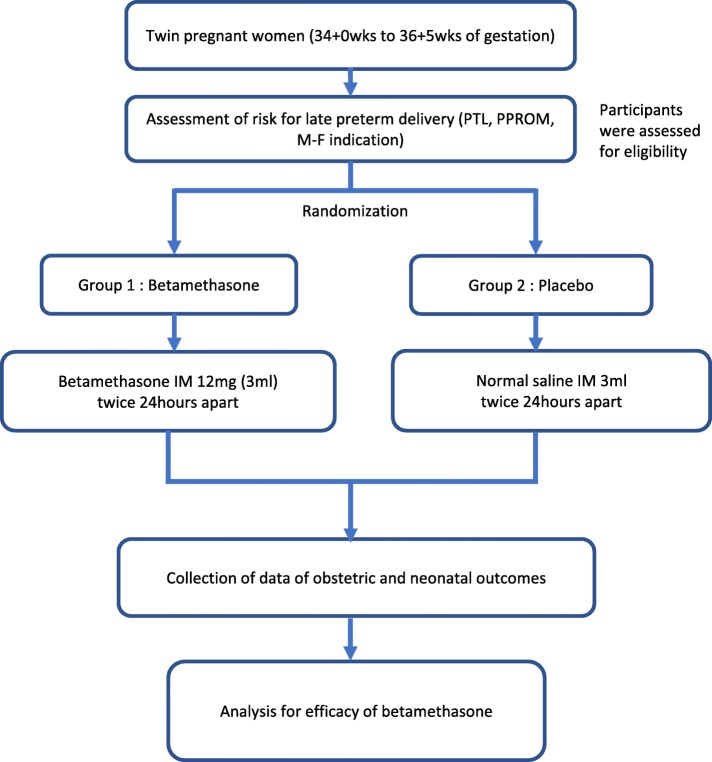
Study flow chart. PTL, preterm labour; PPROM, preterm premature rupture of membranes; M-F indication, maternal foetal indication; IM, intramuscular

In this multicentre, randomized, double-blind placebo-controlled trial, women with a twin pregnancy who are at risk for late preterm birth will be enrolled. Figure 1 shows the model of the study design. After providing written informed consents, the women will be randomly assigned to betamethasone or placebo administration. Except for the administration of antenatal corticosteroids or placebo, the participants will be treated according to obstetric guidelines at the discretion of the attending physician.

### Study setting

This study will be conducted at obstetric departments of two hospitals in South Korea, Seoul National University Hospital and Cheil General Hospital and Women’s Healthcare Centre. They are tertiary referral hospitals and the number of twin delivery is approximately 300–400/year in each centre.

### Study population

Women with a twin pregnancy who are at risk for late preterm delivery will be enrolled at 34 0/7 to 36 5/7 weeks of gestation. The risk for late preterm delivery includes preterm labour with cervical change, preterm premature rupture of membranes, or maternal-foetal indications that require preterm delivery because of maternal (hypertensive disorder, maternal underlying diseases, etc) or foetal causes (oligohydramnios, foetal growth restriction, etc). After screening for eligibility, information regarding the study will be provided and written informed consent will be obtained. Inclusion criteria and exclusion criteria are shown in Table 1. Dropout criteria include patient’s withdrawal of content, occurrence of severe adverse reaction, or clinical situation that does not permit continuation of trial protocol at the discretion of the investigators.

**Table 1:** Inclusion and exclusion criteria

Inclusion criteria	Exclusion criteria
Age > 20 years	Gestational age before 34 weeks 0 days or after 36 weeks 6 days
Women with twin pregnancy at 34 weeks 0 days to 36 weeks 5 days of gestation	Lethal major foetal anomaly, foetal distress or foetal death in utero
At risk for preterm birth	Expected to deliver within 12 h
preterm labour	Advanced cervical dilatation (≥8 cm) in preterm labour
PPROM	Active phase labour (cervical dilatation ≥4 cm) in PPROM
Maternal-foetal indications that need preterm delivery	
Availability of written informed consent	History of a previous administration of antenatal corticosteroid within 1 week
	Administration of systemic steroid for medical indications
	Diagnosis of clinical chorioamnionitis
	Contraindication of betamethasone administration

Preterm labour is defined as regular uterine contractions with or without the following symptoms; pelvic pressure, backache, increased vaginal discharge, menstrual-like cramps, bleeding/show, cervical changes. Clinical chorioamnionitis is defined as fever > 37.8°C and the presence of two more of the following conditions: uterine tenderness, foul-odoured vaginal discharge, maternal leucocytosis(> 1500), maternal tachycardia(> 100) or foetal tachycardia(> 160)

*PPROM* preterm premature rupture of memebrane

### Random assignment method

Enrolled women will be randomly assigned in a 1:1 ratio to antenatal corticosteroids (Group 1) or placebo (Group 2). The randomization will be done by a web-based randomization system that is operated by the medical research collaborating center of Seoul National University Hospital. Unblinded researchers will be designated at the beginning of this trial, including an unblinded pharmacist and unblinded investigators, and they will not participate in the subsequent process of data management and data analysis. The unblinded researchers will prepare the antenatal corticosteroids or placebo according to the treatment assignment. Neither the enrolled pregnant women nor the other investigators (except predeterminate unblinded researchers) will be aware of the result of random assignment.

### Sample size

The sample size was calculated to determine how many neonates will be needed to detect a 30% reduction by antenatal corticosteroids: 20% in the placebo group versus 14% in the antenatal corticosteroids group. We estimated the risk of primary outcome in the placebo group as 20%, with a correlation coefficient between co-twins of 0.32, according to our retrospective twin cohort data. We adopted a risk reduction rate of 30% (from 20 to 14%), according data in previous study [14]. Assuming 80% power, a type I error of 5%, and the ratio of 1:1 between placebo and antenatal corticosteroids, we determined we would require 1616 neonates (808 twin pregnancies).

### Intervention

The antenatal corticosteroids will be betamethasone sodium phosphate 5.2 mg (betamethasone 4.0 mg) in 1 ample (1 mL), produced by Dawon Parm (Korea). Both betamethasone and placebo (normal saline) are colourless liquids. The eligible participants for the inclusion and exclusion criteria will be randomized into two groups: Group 1, antenatal corticosteroids group; Group 2, placebo group. Group 1 consists of pregnant women who will be administered intramuscular betamethasone 12 mg (3 mL) twice in a 24-h interval. Group 2 will be administered the same amount (3 mL) of normal saline twice with the same interval. Once the drug is determined by randomization, the unblinded researchers will prepare and administer betamethasone or placebo to participants.

### Study outcomes

The data on obstetric and neonatal outcome will be gathered. The primary outcome is severe respiratory complications (the use of continuous positive airway pressure or high-flow nasal cannula for at least 12 h, supplemental oxygen administration with a fraction of oxygen 0.3 or more for at least 24 h, mechanical ventilation, or extracorporeal membranes oxygenation) or perinatal death within the first 72 h of delivery. Secondary outcomes are neonatal mortality and/or other neonatal morbidities (Table 2).

**Table 2:** Primary/Secondary outcomes

Primary outcome (within 72 h after delivery)	Secondary outcome (before discharge)
Severe respiratory morbidities	Mild respiratory morbidities
CPAP for ≥12 continuous hours	CPAP for ≥2 continuous hours
High flow nasal cannula for ≥12 continuous hours	High flow nasal cannula for ≥2 continuous hours
Fraction of inspired oxygen of ≥0.3 for ≥24 continuous hours	Fraction of inspired oxygen of ≥0.3 for ≥2 continuous hours
Mechanical ventilation use	Respiratory distress syndrome
ECMO use	Transient tachypnoea of the newborn
Stillbirth	Apnoea
Neonatal death	Bronchopulmonary dysplasia
	Pneumonia
	Surfactant use
	Pulmonary air leak
	Necrotizing enterocolitis
	Intraventricular hemorrhage
	Sepsis
	Hyperbilirubinemia
	Seizures / encephalopathy
	Patent ductus arteriosus
	Hypoglycaemia
	Feeding difficulty
	Need for resuscitation at birth
	Neonatal death
	Maternal complication
	Chorioamnionitis
	Postpartum endometritis

*CPAP* continuous positive airway pressure, *ECMO* extracorporeal membranes oxygenation

### Safety assessment

The patients will be monitored for symptoms in terms of adverse effects. The physicians will check the patients’ vital signs and the occurrence of adverse side effects after injection. Symptoms such as nausea/vomiting, allergic reaction, and local reaction at the injection site will be reported. The administration of the study drug can be interrupted in the occurrence of severe side effects, such as adrenal insufficiency, Cushing’s syndrome, and infection. However, serious side effects after antenatal corticosteroids (betamethasone) were not reported in previous studies in pregnant women [14, 25–27].

### Analysis

The efficacy of betamethasone will be assessed by comparing the primary and secondary outcomes of each group. Efficacy analysis will be conducted based on intention-to-treat and per-protocol principle. Categorical variables will be compared by the chi-square or Fisher’s exact test. For continuous variables, the Mann-Whitney U test will be used. The analysis will be performed by a generalized estimating equation to consider the possibility of the familial correlation between the neonates from a single mother in twin pregnancies [28]. A *p* value of less than 0.05 will be considered significant, and relative risks and 95% confidence intervals will be reported. All analyses will be performed using IBM SPSS Statistics version 23 and R version 3.5.0 (http://www.r-project.org).

## Discussion

With this study, we intend to evaluate the efficacy of antenatal betamethasone in late preterm delivery among twin pregnancies. In clinical practice, antenatal corticosteroids are used to reduce neonatal morbidity and mortality for preterm birth neonates (before 34 weeks of gestation). Recently, the efficacy of the treatment has also been proved for late preterm singleton neonates, and the therapeutic targets of antenatal corticosteroids has been expanded to late preterm neonates (34 0/7 to 36 6/7 weeks of gestation) as well as neonates born before 34 weeks of gestation.

The efficacy of antenatal corticosteroids in twin neonates with early preterm birth (before 34 weeks of gestation) has been demonstrated in several previous reports. However, there has been no prospective randomized trial on the efficacy of antenatal corticosteroids in twin pregnancies in late preterm birth. In this regard, research on this issue is very crucial in clinical practice.

The results of this study are expected to have great impact on several aspects.

First, the result of this study will provide evidence for clinical guidelines of obstetric care in twin pregnancies. To date, there are few obstetric studies on the effectiveness of antenatal corticosteroids in twin pregnancies, and most of the existing studies have been conducted in singleton pregnancies. Because of this, guidelines about the administration of antenatal corticosteroids in twin pregnancy are based on evidences from singleton pregnancy [15, 20].

Second, this study design has methodological strength. This study is a double-blind, randomized controlled study, which provides a high level of evidence. In addition, the objectivity of evaluation variables is expected to yield objective results.

Third, the results of this study will have a significant impact on improving national health and related policies. If the efficacy of antenatal corticosteroids in late preterm neonates is demonstrated, the implementation of guidelines on antenatal corticosteroids in late preterm twin pregnancies will reduce adverse neonatal outcomes and the subsequent burden due to hospitalization of neonates. Otherwise, if the efficacy of antenatal corticosteroids is not proven, it may be the basis for avoiding unnecessary treatment and adverse effects related to corticosteroids.

## Abbreviations

ACOG: American College of Obstetricians and Gynecologists
IM: Intramuscular
NICE: National Institute for Health and Care Excellence
SMFM: Society for Maternal-Fetal Medicine
USA: United States of America
